# Thinking Outside a Less Intact Box: Thalamic Dopamine D2 Receptor Densities Are Negatively Related to Psychometric Creativity in Healthy Individuals

**DOI:** 10.1371/journal.pone.0010670

**Published:** 2010-05-17

**Authors:** Örjan de Manzano, Simon Cervenka, Anke Karabanov, Lars Farde, Fredrik Ullén

**Affiliations:** 1 Neuropediatric Research Unit, Department of Women's and Children's Health and Stockholm Brain Institute, Karolinska Institutet, Stockholm, Sweden; 2 Psychiatry Section, Department of Clinical Neuroscience and Stockholm Brain Institute, Karolinska Institutet, Stockholm, Sweden; 3 Human Motor Control Unit, National Institute of Neurological Disorders and Stroke, National Institutes of Health, Bethesda, Maryland, United States of America; University of Minnesota, United States of America

## Abstract

Several lines of evidence support that dopaminergic neurotransmission plays a role in creative thought and behavior. Here, we investigated the relationship between creative ability and dopamine D2 receptor expression in healthy individuals, with a focus on regions where aberrations in dopaminergic function have previously been associated with psychotic symptoms and a genetic liability to schizophrenia. Scores on divergent thinking tests (Inventiveness battery, Berliner Intelligenz Struktur Test) were correlated with regional D2 receptor densities, as measured by Positron Emission Tomography, and the radioligands [^11^C]raclopride and [^11^C]FLB 457. The results show a negative correlation between divergent thinking scores and D2 density in the thalamus, also when controlling for age and general cognitive ability. Hence, the results demonstrate that the D2 receptor system, and specifically thalamic function, is important for creative performance, and may be one crucial link between creativity and psychopathology. We suggest that decreased D2 receptor densities in the thalamus lower thalamic gating thresholds, thus increasing thalamocortical information flow. In healthy individuals, who do not suffer from the detrimental effects of psychiatric disease, this may increase performance on divergent thinking tests. In combination with the cognitive functions of higher order cortical networks, this could constitute a basis for the generative and selective processes that underlie real life creativity.

## Introduction

Creativity can be generically defined as the ability to produce work that is at the same time novel and meaningful, as opposed to trivial or bizarre [1]. A main psychometric approach to measuring individual differences in creativity is represented by divergent thinking tests. These tests typically involve generating a multitude of novel and meaningful responses to open-ended questions. For instance, in the classical Guilford's alternate uses test [2], participants are instructed to propose different uses for certain artifacts, such as a brick, within a limited time.

Scores on divergent thinking tests show positive correlations with involvement in real-life creative activities, self-rated creativity [3], as well as objective measures of creative achievement, even when controlling for IQ [4]. They are also correlated with several personality traits, such as Openness to Experience, common to individuals with documented creative capacity [3]. In the standard three-stratum model of human cognitive abilities, the Cattell-Horn-Carroll-framework [5], the main ability captured by divergent thinking tests corresponds to the second-order factor “long-term storage and retrieval” (*Glr*), which captures individual differences in fluent retrieval of information through association. This factor, thus, predicts creative achievement over and above fluid reasoning (*Gf*) and crystallized ability (*Gc*), i.e. beyond traditional measures of “intelligence” and “knowledge”.

Divergent thinking is influenced by dopaminergic function. Reuter [6] found a correlation between divergent thinking (the Inventiveness battery of the Berliner Intelligenz Struktur Test) and polymorphisms of the dopamine D2 receptor gene–DRD2 TAQ IA. Higher creativity scores were observed in carriers of the A1 allele. This polymorphism is unrelated to general intelligence [7], [8], which suggests that it is more specifically related to *Glr*. This finding is in line with functional imaging research showing the D2 system to be involved in attentional set shifting and response flexibility, which are important components of divergent thinking [9]. Furthermore, the finding indicates that divergent thinking is related to regional differences in D2 densities, since the DRD2 TAQ IA polymorphism has been shown to modulate D2 binding potential (D2BP) in both striatal [10] and extrastriatal regions [11].

A clue to where to expect regional D2 density differences related to divergent thinking comes from the link between creativity and psychopathology: In healthy individuals, various creativity-related measures, including divergent thinking, have been associated with the personality traits psychoticism and schizotypy, as well as genetic liability for schizophrenia spectrum and bipolar disorders [12]–[21]. Notably, the networks relevant to divergent thinking, i.e. structures and processes in associative corticostriatal-thalamocortical loops [22], [23], overlap to a great extent with regions and networks affected in schizophrenia and bipolar disorder. Furthermore, dopamine is known to influence processing in these networks and alterations in dopaminergic function and activity of D2 receptors have been linked to both positive and negative psychotic symptoms (e.g. [24]–[27]).

Two regions appear to be of particular interest in this context: the thalamus and the striatum. Several studies have shown thalamic D2BP to be reduced in drug-naïve schizophrenia patients [26]–[31]. Moreover, D2BP in subregions of the thalamus was found to be negatively related to total symptoms, general symptoms, positive symptoms, hostility and suspiciousness [28], [31] as well as grandiosity [26]. The direction of this correlation would match with the association between DRD2 TAQ AI and divergent thinking [6]. A metaanalysis by Weinberger and Laurelle [27] found a significant elevation of striatal D2 receptors in untreated patients with schizophrenia. A later study on twins discordant for schizophrenia shows that this upregulation might be related to a genetic risk for schizophrenia [32]. Two studies have found positive correlations between ventral striatal D2BP and the specific symptoms Disorientation [26] and Hallucinations [29].

Although the density of D2 receptors in cortical regions is very low [33] and thus difficult to estimate using PET due to low signal-to noise ratio, a few studies have found correlations between positive symptoms in schizophrenia and D2BP in cortical regions [29], [34], [35]. However, the results across studies are not consistent and more experimental data are required in order to determine their significance to cognition [9]. Hence, it is difficult to state a clear hypothesis about the relation between cortical D2 receptor density and divergent thinking.

Based on these findings, our main hypotheses were that higher scores on divergent thinking would be associated with lower D2BP in the thalamus and/or higher D2BP in the striatum. Since the frontal cortex has been implicated in creative ability from other lines of research (see e.g. [22]), this region was also included in the analysis. For exploratory purposes, we performed an additional analysis of striatal functional subregions.

## Results

**Figure 1:**
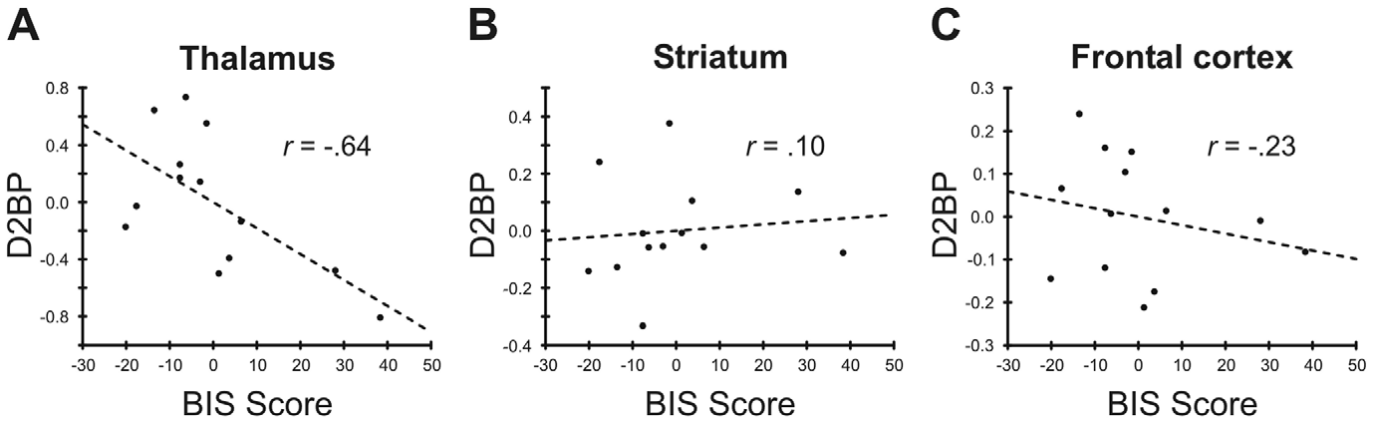
Correlations between divergent thinking scores and dopamine D2 binding potential. (A) Correlation between thalamic D2 binding potential (D2BP) and divergent thinking (BIS score). (B) Correlation between D2BP and BIS score in the striatum. (C) Correlation between D2BP and BIS score in the frontal cortex.

Descriptive statistics of all variables of interest are summarized in Table 1. Table 2 shows partial correlations between regional D2BP and divergent thinking controlling for age. These correlations are also illustrated in Figure 1. There was a significant negative correlation between D2BP and divergent thinking in the thalamus (Fig. 1A; *r* = −.64, *p* = .013; Bonferroni corrected *α* = .017), confirming our hypothesis with regard to this region. This correlation remained significant when simultaneously controlling for age and Raven scores. There was no significant correlation between divergent thinking and D2BP in the striatum (Fig. 1B; *r* = .10, *p* = .37) nor in the frontal cortex (Fig. 1C; *r* = −.23, *p* = .46), or in any striatal subregion (associative striatum: *r* = .05, *p* = .44; ventral striatum: *r* = .35, *p* = .13; sensorimotor striatum: *r* = .07, *p* = .37). Raven scores were not associated with any other measure.

**Table 1:** Mean values, minimum, maximum and standard deviations of all variables of interest.

Measure	Age	BIS	ZF	AM	TN	Raven	Thalamus	Striatum	FC
*M*	59	296	103	98	95	39	2.60	2.63	0.40
Minimum	41	269	83	72	80	29	1.86	2.27	0.21
Maximum	65	350	119	117	114	52	3.28	2.99	0.65
*SD*	8	23	13	15	10	6	0.50	0.18	0.15

Age  =  Participant age; BIS  =  Berliner Intelligenz Struktur Test scores; ZF  =  BIS subtest figural fluency; AM  =  BIS subtest verbal fluency; TN  =  BIS subtest numerical fluency; Raven  =  Raven's Standard Progressive Matrices Plus scores. Thalamus  =  Dopamine D2 receptor binding potential (D2BP) in the thalamus; Striatum  =  D2BP in the striatum; FC  =  D2BP in the frontal cortex.

**Table 2:** Partial correlations between regional D2 receptor binding potential, divergent thinking and intelligence, controlling for age.

Measure	Raven		BIS	
Thalamus	.27	*P* = .40	−.64	*P* = .013* a
Striatum	.39	*P* = .21	.01	*P* = .37^a^
FC	.31	*P* = .32	−.23	*P* = .46

*Significant at p≤0.017, corrected for multiple comparisons (n = 3) using an *α* = 0.05.

a One-tailed *p*-value (direction of correlation according to hypothesis). Other values are two-tailed.

Raven  =  Raven's Standard Progressive Matrices Plus scores; BIS  =  Berliner Intelligenz Struktur Test scores; Thalamus  =  Dopamine D2 receptor binding potential (D2BP) in the thalamus; Striatum  =  D2BP in the striatum; FC  =  D2BP in the frontal cortex.

## Discussion

The main finding in the present study is a negative correlation between divergent thinking and D2BP in the thalamus, thus confirming our hypothesis for this region. There was no significant correlation between divergent thinking and D2BP in the striatum or in the frontal cortex. Furthermore, there were no relations between Raven scores and BIS scores, or between Raven scores and D2BP, which is in line with previous findings showing that divergent thinking is essentially separate from measures of intelligence.

Two methodological issues should be considered. Firstly, the time between the PET-examination and the administration of the divergent thinking test was quite extended (approximately eighteen months). However, in thalamus - where significant results were found - the age effect on D2BP is less pronounced: In a sample of 35 participants, age range 16–50, Talvik [26] found a 6% decline in the right thalamus per decade, and no significant decrease at all in the left thalamus. Secondly, since D2BP is a function of both receptor density and apparent affinity, these parameters cannot be dissociated based on a single PET measurement [36]. Among the factors influencing apparent affinity, endogenous dopamine levels have been shown to affect [^11^C]FLB 457 binding [37], [38], [39]. However, other studies have been negative [40], [41] and using scatchard approaches where Kd and Bmax can be separated is has been shown that Bmax accounts for most of the variance in BP [42]. This suggests that the low D2BP measurements in individuals with high creativity scores demonstrated in the present study are related primarily to a reduced density of dopamine D2 receptors, rather than an increased level of endogenous dopamine.

### Divergent thinking and thalamic D2BP

In the past, several different measures of divergent test performance have been used, e.g. Fluency–the number of valid responses; Originality–how frequent the participant's responses were among the responses of the rest of the sample; Flexibility–the number of semantic categories produced; Switching–the number of shifts between semantic categories; and Elaboration–how extensive each response is (if the task involves producing more than single words). Importantly however, all these measures have been found highly intercorrelated: Someone who has high fluency is also more likely to show more flexibility of thought and provide more elaborate and uncommon solutions. One simple explanation to this was given by Campbell [43] who reformulated an original idea by Guilford [44] into a theory of “blind variation and selective attention”. In brief, if idea generation is based on a more or less random process of free association, and you are able to come up with many ideas, the probability for being flexible and original is consequently increased.

Based on the current findings, we suggest that a lower D2BP in the thalamus may be one factor that facilitates performance on divergent thinking tasks. The thalamus contains the highest levels of dopamine D2 receptors out of all extrastriatal brain regions [33], [45]. Decreased D2BP in the thalamus has been suggested, firstly, to lower thalamic gating thresholds, resulting in decreased filtering and autoregulation of information flow [31] and, secondly, to increase excitation of cortical regions through decreased inhibition of prefrontal pyramidal neurons [46], [47], [48]. The decreased prefrontal signal-to-noise ratio may place networks of cortical neurons in a more labile state, allowing them to more easily switch between representations and process multiple stimuli across a wider association range [49]. This state, which we hereforth will refer to as the “creative bias”, could benefit performance on tasks that involve continuous generation and (re-)combination of mental representations and switching between mind-sets. The creative bias could also explain why the different measures of divergent task performance correlate: A decreased signal-to-noise ratio in thalamus would decrease information gating and possibly increase fluency; decreased signal-to-noise ratio in cortical regions should better enable flexibility and switching between representations; similarly, the associative range should be widened and selectivity should be decreased which might spur originality and elaboration.

### Divergent thinking and psychopathology

Besides carrying benefits related to fluency and switching, the decreased signal-to-noise ratio associated with the creative bias should be disadvantageous in relation to tasks that require high levels of selective attention. Some support for this prediction can be taken from Dorfman [50] who showed that the greater a person's divergent thinking scores, the slower his or her reaction times were on a negative priming task requiring the inhibition of interfering information. Furthermore, the creative bias may also bring a risk of excessive excitatory signals from the thalamus overwhelming cortical neurotransmission, with ensuing cognitive disorganization and positive symptoms [30]. It is thus tempting to suggest that dopaminergic modulation of neurotransmission mediated through dopamine D2-receptors could be one of the mechanisms which associate creativity with positive psychotic symptoms. Interestingly, positive symptoms are not necessarily related to problems in executive function, at least not to the same extent as negative symptoms [51], which indicates that in the creative individual “blind variation” might be affected without a concomitant decline in “selective retention”. It can be speculated that aberrant thalamic function may promote unusual associations, as well as improved performance on divergent thinking tests in healthy individuals, in the absence of the detrimental effects typically associated with psychiatric disorders. In other words, thinking outside the box might be facilitated by having a somewhat less intact box.

## Materials and Methods

### Ethics statement

All participants gave verbal and written informed consent and the study was approved by the Ethics and Radiation Safety committees of Karolinska Institutet (Dnr. 2007/704-31/4, 02-431, 2007/1611-32).

### Participants

The participants in the present study had previously served as control subjects in a clinical study [52]. Fourteen participants (6 male, 8 female) with at least nine years of basic education were included in the study (age: 41–65, M = 56±8 years). The participants had no history of neurological or psychiatric illness, as determined by clinical interview, MRI examination, blood and urine tests, and ECG. None of the subjects were nicotine users, and the use of caffeine or alcohol was not allowed during the days of PET examinations. All women were menopausal. One participant was excluded from analysis because of an extremely low Raven score, giving a total of thirteen participants.

### Psychological measurements

The psychological tests were administered individually. The personality questionnaires were administered in connection with the PET-experiments. Divergent thinking and general cognitive ability was assessed approximately eighteen months after the original PET-examination.

#### Divergent thinking

Divergent thinking was assessed using three timed subtests from the “inventiveness” test battery of the “Berliner Intelligenz Struktur Test” (BIS) [53]. The tests measured performance within the figural, verbal, and numeric domains and were chosen based on their having the highest factor loadings on the total inventiveness-score. In the figural test, a simple line drawing should be completed in various ways in order to create pictures of as many possible real objects as possible. In the verbal test, the participant was instructed to produce as many alternate uses for a given object as possible. In the numeric test the participant had to generate as many logical number sequences as possible, while trying to vary the rule of construction. Raw scores from each subtest were transformed into Z scores [53], which were subsequently summed and used as a composite measure of divergent thinking.

#### General cognitive ability

General cognitive ability was assessed using the Raven's Standard Progressive Matrices Plus (Raven) [54], a widely used test that mainly reflects psychometric general intelligence (*g*) [55]. The test was administered without time limit.

### MR and PET experimental procedure

#### Magnetic resonance (MR) and head fixation system

T1 weighted MR-images were acquired using a 1.5 T GE Signa system (Milwaukee, WI) To allow for the same head position in all measurements and to minimize head movement, a plaster helmet was made for each participant individually and used during both MRI and PET examinations [56].

#### Positron emission tomography (PET) examinations

PET studies were performed on an ECAT Exact HR system (CTI Siemens, Knoxville, TN) [^11^C]raclopride and [^11^C]FLB 457 were prepared from [^11^C]methyl triflate as described previously [57], [58]. The radioligands were given intravenously as a rapid bolus and the cannula was flushed with saline. Radioactivity in the brain was measured during 51 min for [^11^C]raclopride and 87 min for [^11^C]FLB 457,

#### Image processing and analysis

The MR-images were realigned to the AC–PC plane using the SPM2 software and PET images were coregistered to the MR image using the normalized mutual information method implemented in SPM2 (Wellcome Department of Imaging Neuroscience, London, UK). For determination of regional radioligand binding, regions of interest (ROIs) were manually delineated on each individual MR-image using the Human Brain Atlas software.

**Figure 2:**
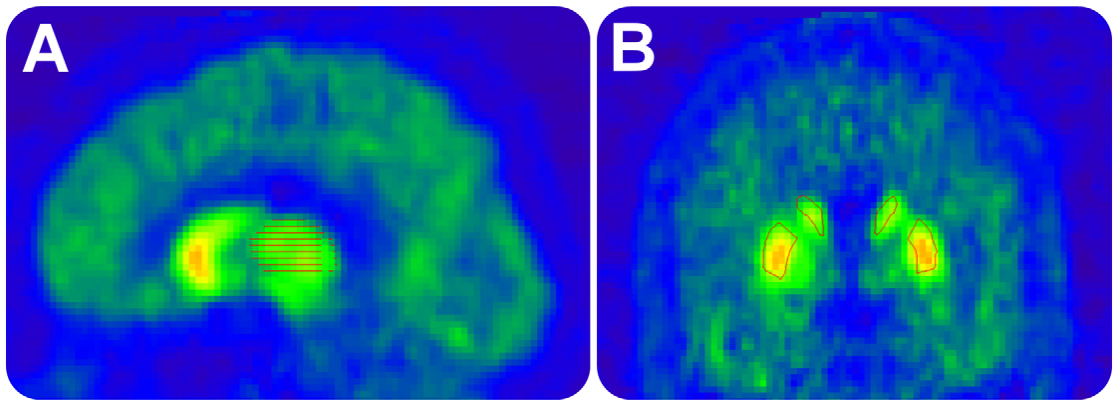
Regions of interest (ROI). (A) Sagittal image of regional radioactivity after intravenous injection of [^11^C]FLB 457 in one subject. Thalamic ROI. (B) Coronal image of regional radioactivity after intravenous injection of [^11^C]raclopride. Striatal ROI.

In the present study, the selection of ROIs was limited to the thalamus and frontal cortex for [^11^C]FLB 457 examinations, and to the striatum for [^11^C]raclopride examinations. In an extended anatomical analysis of the striatum, ROIs for striatal subregions were defined according to a method described in the literature [59], [60] in which striatum is divided into ventral, associative and sensorimotor subregions based on the differential connectivity of the striatum [61]. ROIs for the thalamus were defined using a modified version of a procedure described previously [31], [62], [63]. Finally, a ROI for cerebellum was drawn below the petrosal bone using five slices, corresponding to a thickness of 10 mm. For an example of ROI delineation, see Figure 2.

The ROIs were transferred to the series of PET images to generate time–activity curves (procedure described elsewhere [52]). D2 receptor binding potential (BP) values were calculated using the simplified reference tissue model with the cerebellum as reference region. In this context, BP refers to BP_ND_, which represents the ratio at equilibrium of specifically bound radioligand to that of nondisplaceable radioligand in tissue [64]. The SRTM has previously been validated for both [^11^C]raclopride and [^11^C]FLB 457 [65] and [66].

### Data analysis

Data was analyzed using Statistica 8.0 (StatSoft). A partial correlation between regional thalamic D2BP, striatal D2BP, intelligence and divergent thinking was then performed, keeping participant age constant. For the thalamus and the striatum, one-tailed significance tests were used, since we predicted negative and positive directions of the correlations, respectively. Significance levels were Bonferroni corrected (n = 3; thalamus/striatum/frontal cortex). Striatal subregions were analyzed separately in an extended analysis, also using one-tailed significance tests.
